# Combustion of High-Energy Compositions (HECs) Containing Al-B, Ti-B and Fe-B Ultrafine Powders (UFPs)

**DOI:** 10.3390/nano15070543

**Published:** 2025-04-02

**Authors:** Weiqiang Pang, Ivan Sorokin, Alexander Korotkikh

**Affiliations:** 1 Xi’an Modern Chemistry Research Institute, Xi’an 710065, China; nwpu_pwq@163.com; 2 Voevodsky Institute of Chemical Kinetics and Combustion of the SB RAS, Novosibirsk 630090, Russia; ivans3485@gmail.com; 3 School of Energy and Power Engineering, Tomsk Polytechnic University, Tomsk 634050, Russia; 4 Research Institute of Applied Mathematics and Mechanics, Tomsk State University, Tomsk 634050, Russia

**Keywords:** ultrafine metal, high-energy composition, ignition, combustion, heat release, kinetics, burning rate

## Abstract

Metal and metalloid powders are widely used in high-energy compositions (HECs) and solid propellants (SPs), increasing their energetic characteristics in the combustion chamber. The particle size distribution, protective coatings of the particles and heat of combustion of the metal powders influence the ignition and combustion parameters of the HECs as well as the characteristics of the propulsion systems. Boron-based metallic fuels achieve high-energy potentials during their combustion. The effect of Al-B, Fe-B and Ti-B (Me-B) mixture ultrafine powders (UFPs) on the ignition and combustion characteristics of a model HEC based on a solid oxidizer and a polymer combustible binder was investigated. The Me-B mass ratios in the mixture UFPs corresponded to the phase composition of the borides AlB_2_, FeB and TiB_2_. It was found that replacing the aluminum UFP with Al-B, Fe-B and Ti-B UFPs in the HECs changed the exponent (*n*) in the correlations of the ignition delay time *t*_ign_(*q*) and burning rate *u*(*p*). The maximum burning rate and *n* over the pressure range of 0.5–5.0 MPa were obtained for the HEC with Al-B UFPs due to the increase in the heat release rate near the sample surface during the joint combustion of the Al and B particles.

## 1. Introduction

### 1.1. Ultrafine Powders of Metal

Ultrafine metals and metalloids (such as lithium, beryllium, magnesium, aluminum, titanium, iron, nickel, copper, boron, silicon and their alloys with various crystal structures) are used in a wide range of industries, including non-ferrous metallurgy, aerospace, microelectronics, energy, automotive, chemical, medical and military [1,2,3,4]. Their unique properties make them indispensable for the creation of high-performance and durable materials (hard alloys, ceramic, magnetic and composite materials), additives to lubricants, superconductors, filters, hybrid fuels (HFs) and solid propellants (SPs) [5,6,7,8]. There are several advantages of ultrafine powders (UFPs) over micron powders, including improving mechanical properties, high specific surface areas (SSAs) and reactivities, lower ignition temperature, melting and evaporation temperatures, ensuring a high combustion completeness and a uniformity of heat released in the production of ceramic and synthetic materials. However, their energetic application is limited by their disadvantages, including a tendency to aggregate particles, pyrophoricity and storage and transport complexities.

There are several methods for producing UFPs of metals, metalloids and their compounds that are used on laboratory and industrial scales. The particles’ shape and size, the SSA and the active metal content depend on the production methods (mechanical, physical or chemical) [9,10,11]. The mechanical methods are based on the application of pressure, friction, vibration and other mechanical effects on the material. Ball milling, vibrating ball milling and horoscope milling are commonly used. The particles’ shape obtained in ball milling is irregular and their surface is smooth. Gas-jet milling provides a finer grinding of the basic material when the action of a gas jet under pressure (air, inert gases) is fed into the working chamber through nozzles at the speed of sound or at supersonic speed. In the chamber, the initial particles collide with a metal wall or with each other. In jet milling, for example, porous metal structures obtained by electrochemical methods can be ground. The fraction of fine particles can be increased by applying cryogenic grinding technology. The physical methods for producing metal UFPs are based on the evaporation and subsequent condensation of the elements and their compounds. Heating and evaporation are carried out by plasma, laser, electric arc discharge, ionization, high-frequency currents or electron irradiation. The supersaturated steam is created by evaporating the material from a heated melt surface which is quickly cooled by the jet supply of coolant, flowing through nozzles and expanding. When cooled, the vapor droplets are solidified to form an aerosol system containing solid particles that can be captured by various methods. One of the main methods for producing metal UFPs, which produces nano-sized particles in large quantities, is the method of electrical explosion of a metal wire [12,13] placed between two electrodes. This method is also used for producing bimetallic nano-sized particles [14], which expands the applications of UFPs. The electrical explosion of a wire (EEW) is carried out by supplying a powerful high-voltage nanosecond electric current pulse with a density of 10^6^–10^9^ A cm^−2^ to a conductor (a metal wire with a diameter of 0.1–1.0 mm). The wire is heated to a melting point, melted and then destroyed by an explosion. When the explosion products expand, nanoparticles are formed in the gas atmosphere. The ultra-fast quenching of the electrical explosion products of a conductor at a rate of up to 10^7^ K s^−1^ provides powders with particle sizes less than 0.5 μm. The chemical methods for producing metals (or oxides) are mainly based on reducing chemical reactions, catalytic and thermal decomposition of metal-organic compounds, hydrides or metal salts, electrolysis of metal salts and solvothermal and molten salt synthesis [15,16]. These methods allow the synthesis of nano-sized particles over a narrow size range and with high product purity, but they are expensive.

### 1.2. Use of Metal UFPs in SPs and HECs

Incorporating metal UFPs into SPs, HFs and high-energy compositions (HECs) presents significant benefits in heat release efficiency, ignition and combustion characteristics. The heat of combustion is an important characteristic in the context of metal use in HECs and SPs. Boron (B) and aluminum borides (AlB_2_, AlB_10_, AlB_12_) have high heat of combustion per unit mass or volume (~58.1 kJ g^−1^ and 43.0–53.5 kJ g^−1^ or ~136.0 kJ cm^−3^ and 122.0–135.9 kJ cm^−3^, respectively) with a complete oxidation of particles, which makes it the most energy-efficient metal [17,18,19,20,21,22,23]. The high-energy potential of B powder is difficult to achieve in fast-reacting fuel systems due to the presence of an oxide layer (B_2_O_3_) on the particles’ surface, causing slow and incomplete combustion. One of the main reasons for incomplete B combustion is slow thermal oxidation due to the formation of a liquid B_2_O_3_ layer on the particles’ surface, which reduces the diffusion rate of the oxidizer in the B core. The thickness of the B_2_O_3_ cover decreases during its evaporation (1860 °C) and heterogeneous surface reactions. The oxidizer from the environment diffuses through the molten B_2_O_3_ and B diffuses from inside the particle, which is a complex chemical and physical process. The oxidation of the B particle occurs in several stages, starting with the formation of the intermediate gaseous products BO and BO_2_, which diffuse outward. In a hydrogen- or steam-containing gas medium, the heat of combustion of B is low due to the formation of boric acid (HBO_2_) [18,19,24,25,26]. The decrease in particle size in UFP B reduces the ignition delay time (*t*_ign_) and the ignition temperature (*T_ign_*) during intensive heating, increasing the degree of B oxidation [20]. Aluminum (Al) is the most common component due to its mid-level heat of combustion and density, relative safety, absence of toxic combustion products and low cost. Therefore, Al powders are widely used in SPs and pyrotechnic, explosive and high-energy systems. The heat of combustion of Al is ~31.0 kJ g^−1^, slightly higher than the heat of combustion of magnesium (Mg) and similar to that of carbon (C) (~27.0 kJ g^−1^ and 32.8 kJ g^−1^, respectively). However, the density of Al is 2.70 g cm^−3^ (at 20 °C), which is higher than that of Mg (1.74 g cm^−3^) and C (2.27 g cm^−3^), so the volumetric heat of combustion is higher than the others (83.7 kJ cm^−3^). Al combustion occurs in a gaseous oxidizing medium, which contributes to its increase in temperature and the rate of chemical reactions near the fuel or SP surface. The *T_ign_* and burning time (*t*_b_) of Al depends on the particles’ size, the passivation coating on the particles’ surface and the temperature, pressure and composition of the gaseous medium. The oxide coating slows down the intensity of ignition and the combustion processes of powder. In the equation of the ignition delay time *t*_ign_(*q*) = *a* · *q*^−n^ for the heat flux density (*q*), the exponent (*n*) for Al and AlB_n_ micron powders is equal to ~2.0. For aluminum (Alex) and B UFPs, the *n* is significantly smaller (1.5 and 1.0, respectively) due to rapid heating and a possible change in the ignition mechanism of Alex and B UFPs. The *t*_ign_ and the ignition energy for Alex and B UFPs are less dependent on the heat flux and significantly less (6–10 times) than those of micro-scale metals under intensive laser heating (65–190 W cm^−2^ in air). The combustion of micro-sized aluminum (μAl) particles is satisfactorily described by a generalized correlation of the burning time, *t*_b_(*D*) = *c* · *D^n^* · *X*_eff_^−1.0^ · *p*^−0.1^ *T*^−0.2^, to the particle initial diameter (*D*) and the effective oxidizer concentration *X*_eff_ = *C*_O2_ + 0.6*C*_H2O_ + 0.22*C*_CO2_ as well as the pressure (*p*) and temperature (*T*) of the gas medium, obtained on the basis of more than 400 experimental values of the burning time of μAl particles, with the indicators *c* = 0.00735, *n* = 1.80 (M. Beckstead) and *c* = 0.00213, *n* = 1.72 (A. Braconnier). The burning time of Alex is slightly dependent on the initial particle diameter (*n* = 0.15–0.34). It is obvious that the pressure, temperature and oxidizer concentration of the gas medium have a greater influence [6,20,27,28,29,30,31,32]. When igniting Alex (with a heating rate above 10^6^ K s^−1^) or when burning Al, Al-Li and Al-Mg agglomerate particles, the melting and dispersive boiling mechanism of the droplet core (microexplosive combustion of the particle) can be realized [33,34,35,36], which contributes to the particles’ size reduction, the reduction of condensed combustion products (CCPs) and the more efficient combustion of SPs in the combustion chamber. Therefore, the study of the kinetics, mechanisms and completeness of combustion of Al particles under different conditions is still relevant and has practical significance.

A small amount of additives (up to 2 wt.% of B, Al, titanium (Ti), iron (Fe), nickel (Ni), copper (Cu), zinc (Zn), molybdenum (Mo) and tungsten (W) powders or their oxides) catalyzes the combustion process for HECs based on AP [11,37,38,39]. The most effective additives are UFPs of Fe, Cu and Zn. The introduction of Fe, Ni, Cu, or Zn UFPs has been found to reduce the *t*_ign_ of HECs and the power exponent in the correlations of the *t*_ign_(*q*) and *u*(*p*) = *b* · *p*^n^ on pressure. With an increase in the SSA of the metal powder, the number of active centers and oxygen vacancies on the surface of the particles increases sharply, which intensifies the thermal decomposition of AP and the combustion process of the HECs. Metal borides, such as AlB_2_, AlB_12_, FeB and TiB_2_, also intensify the physical and chemical processes and increase the *u* of HECs and HFs [18,19,20,21,22,37,40,41,42,43].

In this work, the effect of Al-B, Fe-B and Ti-B (Me-B) mixture UFPs on the ignition and combustion characteristics for a model HECs based on a solid oxidizer and a polymer combustible binder were carried out. The mass ratios of the Me-B mixture in the UFPs corresponded to the phase composition of the borides AlB_2_, FeB and TiB_2_.

## 2. Materials and Methods

### 2.1. Ultrafine Powders of B, Al, Fe and Ti

Mechanical mixtures of UFPs of Al-B (mass ratio of 55.5/44.5%), Fe-B (83.7/16.3%) and Ti-B (68.9/31.1%) were used as high-energy metal fuels. Commercial Al, Fe and Ti powders were obtained by an EEW method in argon and passivated (“Advanced Powder Technologies” LLC, Tomsk, Russia). Amorphous B powder B-99A was produced in “Aviabor” LLC (N. Novgorod, Russia). All powders had a protective coating on the particle surface, therefore Alex and B powders were stored in air, and Ti and Fe powders were stored in liquid hexane C_6_H_14_. The protective layer on the particles’ surface is usually formed by controlled oxidation of the metal particles (in an argon and oxygen environment). In some cases, nitrogen can be used for passivation of Ti nano-scale particles to create a nitride surface layer or wetting with liquid hexane for the protective layer formation.

### 2.2. Ignition of HECs with Me UFPs

The model HEC samples contained 64.6 wt.% AP of two grades (particle size less than 50 μm (0.6 mass fraction) and 160–315 μm (0.4 mass fraction)), 19.7 wt.% SKDM-80 butadiene rubber and 15.7 wt.% metal UFP. The production of mixed HECs was carried out by mixing and continuous pressing. The ignition characteristics of the HECs containing metal UFPs with a sample diameter of 10 mm and a height of 5 mm were measured by using an experimental testbed in which a continuous CO_2_ laser with an adjustable power from 10 to 200 W was used as an external heating source [44]. The thermal power and heat flux density on the sample surface was measured with a thermal laser power sensor (FL400A-BB-50, Ophir Optronics Solutions). The thermal decomposition onset time of the HECs was measured with the reactive force sensor of the outflow of gas products from the sample surface when it was heated by laser. The *t*_ign_ of the HECs was determined by the difference in electrical signals from the photodiodes, recorded via an analog-to-digital converter (L-card E-14-440) on a laptop at the beginning of the heating of the HEC sample and at the time of the appearance of glow and flame near the sample surface. The ignition criterion for *t*_ign_ measurement was the beginning of the formation of a visible flame zone, whose propagation lead to self-sustaining combustion of the HECs when the laser radiation was switched off.

### 2.3. Combustion of HECs with Me UFPs

The steady burning rate (*u*) of the HEC samples with a diameter of 10 mm and a height of 30 mm at excess pressures of 0.5–5.0 MPa was measured by using the burning-wire method and a constant-pressure bomb with nitrogen [45]. Measured with an oscilloscope, the burning time of a known sample height was used to calculate the *u*. Before the experiment, the lateral surface of the cylindrical HECs were reinforced with a double layer of insulating tape. The outflow of gases and CCPs occurred on the end surface of the HECs, which made it possible to capture condensed products using sieves and filters.

## 3. Results and Discussion

### 3.1. SEM and Particle Size of UFPs of B, Al, Fe and Ti

**Figure 1:**
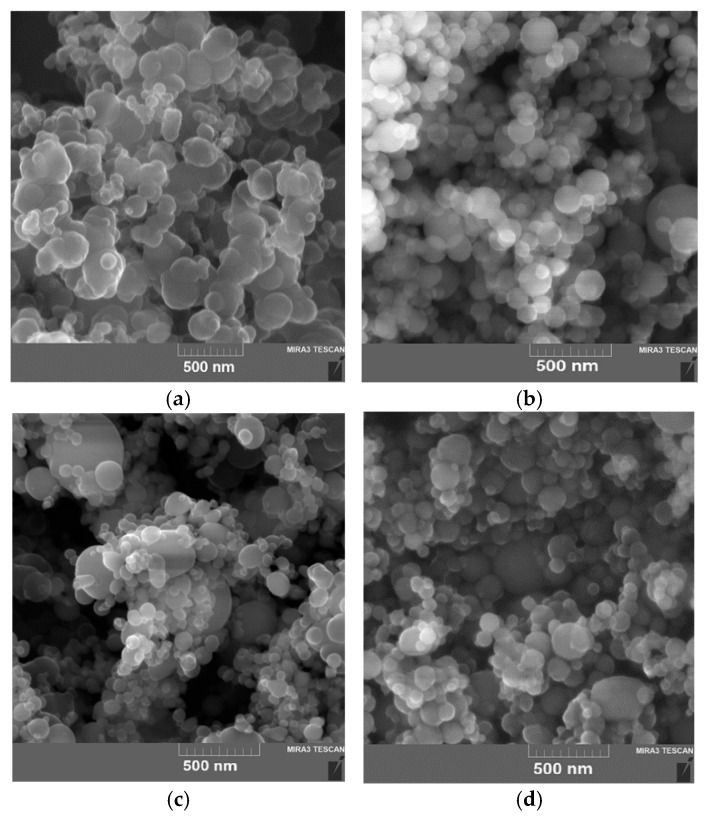
SEM images of UFPs: (**a**) amorphous B; (**b**) Al (Alex); (**c**) Ti; (**d**) Fe.

The SEM images of B, Al, Ti and Fe powders obtained using a TESCAN MIRA 3 LMU, Brno, Czech Republic, scanning electron microscope (SEM) are presented in Figure 1. The powders used had spherical particles with a protective layer. Boron particles tend to aggregate and form larger micro-sized particles (1–2 μm) with a high SSA of 9–15 m^2^ g^−1^. The average diameter and SSA of the B particles were 210–230 nm and 8.6 m^2^ g^−1^ and for Al, Fe and Ti were 90–110 nm and 15.5, 7.7 and 13.8 m^2^ g^−1^, respectively (the manufacturer’s data).

### 3.2. The t_ign_ of HECs with Me UFPs

**Figure 2:**
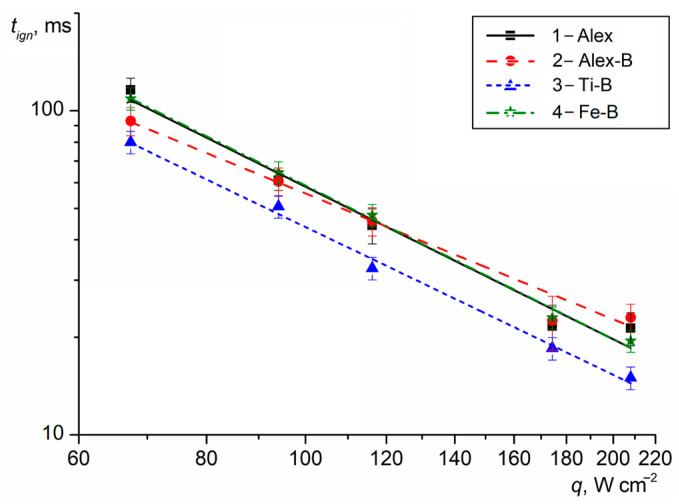
The *t_ign_* of the HECs with Me UFPs vs. the heat flux density.

The thermal decomposition onset time, the *T*_ign_ and *t*_ign_ for the HECs with Me UFPs were determined at the fixed heat flux density *q*. The heat flux density range used for the end surface sample heating was *q* = 65–210 W cm^−2^. The *T*_ign_ of HECs, measured using a Jade J530 SB thermal imaging camera, was over the temperature range of 350–650 °C and mainly depended on the heating rate and heat flux density of the laser radiation. The measured *t*_ign_ of the HECs (points) as a function of the heat flux density are presented in Figure 2. The lines represent the generalized correlations *t*_ign_(*q*) = *a* · *q*^−*n*^.

Note that the *t_ign_* for HEC-1 with Alex decreased from 116 to 18 ms as *q* increased from 65 to 210 W cm^−2^. The *n* exponent in *t*_ign_(*q*) was at its maximum and equal to 1.60. At *q* = 67 W cm^−2^, the period of time between heating the sample surface to ~340 °C and the beginning of the formation of gas products during the HEC-1 thermal decomposition was ~60 ms. When the sample surface was heated to ~400–420 °C (*t* = 100–105 ms), the outflow rate of the gas products increased and a luminous spot with a diameter of 3 mm was formed on the sample surface. After 1–1.5 ms, the ignition of the gas products with Al particles was recorded with the subsequent formation of a flame zone (*t* = 108–112 ms) near the sample surface. The maximum flame propagation rate was ~8.5 m s^−1^. The Al particles with a high SSA on the HEC-1 sample surface intensified the physical and chemical processes due to the additional heat release during their oxidation, the increase in the rate of the oxidation reactions in the flame zone and the reduction in the time to the steady burning of the sample. Moreover, when the burning Al-agglomerated particles were expelled from the flame zone into the air and their surface was sharply cooled, a thin oxide layer was formed, which increased the excess pressure in the encapsulated core (accumulation of gaseous AlO and AlO_2_ suboxided at the boundary of the inner surface of the oxide layer) and the probability of the micro-explosive combustion of the particles in the final stage [36]. The burning time of the Al particle fragments was significantly reduced due to a decrease in their particles size and the increase in the SSA.

When Alex was replaced with Me-B UFP in the HECs, the *t*_ign_ of the samples changed over the entire range of *q* under consideration. However, the greatest efficiency in reducing the *t*_ign_ was achieved by HEC-3 with Ti-B, for which the *t*_ign_ values were reduced by 20–27% compared to the base HEC-1 with Alex. At the same time, the exponent *n* was reduced to 1.52. At *q* = 67 W cm^−2^, the time to reach a sample surface temperature of 340 °C was reduced to ~50 ms and after 15 ms, a luminous spot on the HEC-3 surface with a subsequent outflow of gas products, including Ti and B burning particles (*t* = 66–72 ms), was recorded. When the sample surface was heated to 490 °C (~78–80 ms), the appearance of a flame was registered near the HEC surface. The propagation rate of the flame zone was ~6.5 m s^−1^. The fast ignition and micro-explosive combustion of Ti particles with a characteristic star formation (similar to those observed for micro-size particles [46,47]) promotes the thermal oxidation of B particles and the development of flame processes with increasing temperature and gas outflow rate from the HEC surface.

The use of Al-B UFPs in HEC-2 reduced the *t*_ign_ by 6–16% over the heat flux density range of 65–100 W cm^−2^ compared with the *t*_ign_ of HEC-1. As the values of *q* increased up to 150–210 W cm^−2^, the *t*_ign_ increased by 5–14%. The exponent *n* in *t*_ign_(*q*) was at a minimum and equal to 1.34. The heating time of the sample surface layer to a temperature of 340 °C was ~56 ms. The flame appearance was recorded after ~37 ms. The rate of flame zone propagation reached 6.5 m s^−1^. Reducing the concentration of Al particles by replacing them with B particles on the sample surface increased the rate of flame formation and the achievement of the stable combustion of HEC-2 at *q* < 120 W cm^−2^.

In the case of using Fe-B UFPs in HEC-4, the ignition time had similar values (difference was less than 3%) in comparison to the *t*_ign_ of HEC-1 with Alex. The *n* exponent was 1.57. The heating time of the surface layer to a temperature of 340 °C was ~50 ms. When a luminous spot appeared on the sample surface, an intense stream of thermal decomposition gas products (*t* = 65–70 ms) with small particles from the surface were recorded. The flame formation near the surface of the HEC-4 sample (*t* = 100–105 ms) occurred when the surface was heated to a temperature of 430 °C. However, the maximum propagation rate of the flame zone was ~8.6 m s^−1^. Iron particles with an oxide cover catalyzed the thermal decomposition of the oxidizer crystals and increased the outflow rate of gas products from the sample surface, forming a flame zone with the maximum length and high-temperature process. Additional heat release during the oxidation of the B particles in a flame increased the rate of the chemical reactions and the HEC-4 combustion stability.

Thus, the ignition efficiency by using Me-B UFPs in HECs can be represented as a series: at *q* = 65 W cm^−2^, [*t*_ign_ HEC-3, Ti-B] < [*t*_ign_ HEC-2, Al-B] < [*t*_ign_ HEC-4, Fe-B] ≈ [*t*_ign_ HEC-1, Alex] and at *q* = 210 W cm^−2^, [*t*_ign_ HEC-3, Ti-B] < [*t*_ign_ HEC-1, Alex] < [*t*_ign_ HEC-4, Fe-B] < [*t*_ign_ HEC-2, Al-B].

### 3.3. Ignition Kinetics of HECs with Me UFPs

The kinetic parameters of reactions in the HEC condensed phase (activation energy (*E*) and pre-exponential factor (*z*)) were obtained at high heating rates of the HEC surface based on the experimentally determined *t*_ign_ at given initiating *q* [48]. These parameters are necessary in calculating the time and energy characteristics of ignition and combustion of HEC, provided that the main reactions in the sample reaction layer take into account a set of reaction mechanisms that can change with variation in the heating rate, pressure, reactants physical state and other conditions. Moreover, with rapid heating of the surface (more than 1000 K · s^−1^) and combustion of the HEM in the high-temperature reaction zone, as a rule, physical and chemical processes are accompanied by melting, evaporation and diffusion mass transfer. These processes are characterized by their own time, which should also be taken into account when determining the boundaries of the kinetic parameters. The *t*_ign_(*q*) correlation and the calculation method of the formal kinetic parameters of ignition [38] are applied to identify *E*, the thermal effect of the exothermic reactions with *z* (*Q* · *z*), the ignition temperature *T*_ign_ on the sample surface and also the heat release rate (*W*) in condensed phase when the HECs is exposed to a radiant heat flux (Table 1). The *T*_ign_ is calculated analytically based on experimentally determined *t*_ign_ at given *q*, the thermal conductivity coefficient (*λ*), specific heat capacity (*c*), and density (*ρ*) for HECs. Replacing Alex with Al-B, Ti-B and Fe-B UFPs in HECs causes a significant decrease in the heat release rate in the thermal layer surface (up to 30–35%), while the *T_ign_* on the reaction surface remains in the range *T*_ign_ = 461–566 K, depending on the active heat flux *q*.

### 3.4. The u of HECs with Me UFPs

**Figure 3:**
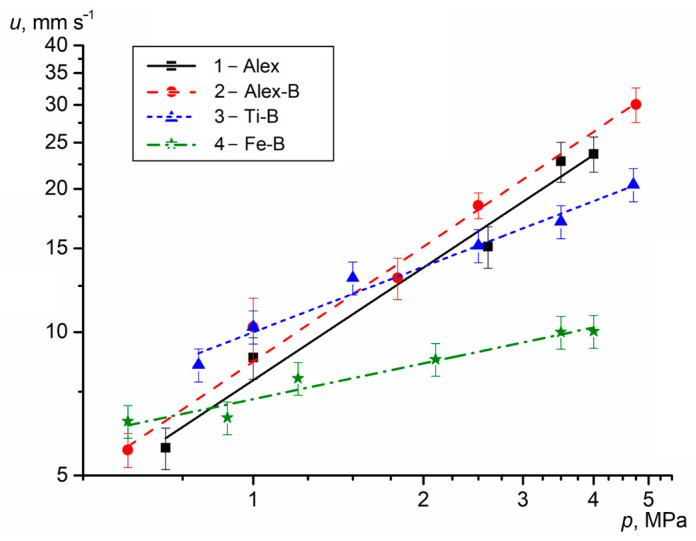
The *u* of the HECs with Me UFPs vs. the pressure.

The *u* is an important characteristic for HECs and SPs affecting the outflow rate of combustion products from the HECs surface and the outflow rate of combustion gas products from the engine nozzle. The *u* of the HECs is measured at different excess pressures *p* in a constant-pressure bomb. The average measured values (points) of the *u* for the HECs with Me UFPs as a function of pressure are presented in Figure 3. The experimental data were used in obtaining the burning rate law as *u*(*p*) = *b* · *p^n^*.

The *u* of the HEC-1 with Alex increases from 4.7 to 27.5 mm s^−1^ with the chamber pressure rising from 0.5 to 5.0 MPa, while exponent *n* in *u*(*p*) correlation is equal to 0.77. The use of Al-B UFP in the HEC-2 increases the *u* by 7–18% (as a *p* function), but *n* is the maximum and equal to 0.81. Replacing Alex with Ti-B UFP in the HEC-3 causes an increase in the *u* by 10–55% over the pressure range of 0.5–1.5 MPa and decrease in the *u* by 6–24% over the pressure range of 2.5–5.0 MPa due to decreasing exponent *n* to 0.45. Note that the minimum exponent *n* = 0.25 is obtained for the HEC-4 with Fe-B UFP, the *u* of which is lower than that of the base HEC-1 with Alex by 9–61% as a function of pressure. However, at a low pressure (*p* = 0.5 MPa), the *u* of HEC-4 is 6.1 mm s^−1^ and 30% higher than that for *u* base HEC-1.

Thus, the use of Al-B UFP in the HEC-2 increases the *u* to 5.0–32.3 mm s^−1^ with a pressure change from 0.5 to 5.0 MPa due to the high reactivity, intense energy release of Al particles and an increase in the burning rate of B particles in the reaction layer and gas decomposition products near the combustion surface. A comparative analysis with published data of the *u* for the same component HEC compositions with micro-sized AlB_2_ and AlB_12_ powders [21] show that the *u* of HEC-2 with Al-B UFP is 1.4–3.7 times higher and the exponent *n* is 80% higher at the same pressure range. The use of highly reactive Al and B nano-sized particles increases their oxidation and heat release degree, and the temperature on the HEC combustion surface. Accordingly, the HEC-2 with Al-B UFP has greater sensitivity to pressure change in the combustion chamber. The use of Ti-B and Fe-B UFPs in the HEC-3 and HEC-4 increases the *u* to 7.3 mm s^−1^ (by 55%) and 6.1 mm s^−1^ (by 30%) at *p* = 0.5 MPa (in compare with *u* HEC-1). However, the combustion efficiency for these HECs decreases with increasing chamber pressure due to the possible relative reduction of the heat release rate and temperature on the HEC reaction layer surface.

As previously noted, the metal and B combustion is a multi-stage physical and chemical process that depends on the initial diameter and protective coating of the particles, temperature, pressure and oxidizer concentration of the gas medium. The combustion mode of metals may differ depending on external conditions, the boiling point of the metal and the evaporation temperature of the oxide coating of the particles. The combustion of Al particles occurs in the diffusion mode during the destruction or melting of the oxide coating and the active aluminum evaporation. Their combustion intensity mainly depends on the initial particle diameter and the thickness of the oxide coating. The high reactivity of the Al nanoparticles increases the heat release rate in the condensed phase (Table 1) and the burning rate of the HEC (Figure 3). An *p* increase in the chamber helps to increase the *u* of the HEC, reduce the width of the chemical reaction zone and the reaction time of metal particles with reagents on the surface. Particles of Ti, Fe and B burn more slowly in the heterogeneous mode due to their high boiling and evaporation temperatures. An increase in the SSA and possible micro-explosive combustion of particles (in the final stages Ti or under certain conditions Fe) contributes to the intensification of heterogeneous reactions on the surface and a decrease in the burning time of the particles. With temperature increase on the particle surface, the evaporation rate of the oxide coatings and the diffusion of the gas oxidizer increases, which intensifies the burning process and increases the completeness of particle combustion. When replacing Alex with Ti, Fe or B, the heat release rate in the reaction layer of the HEC is reduced by 40–50% (Table 1), while the *u* of propellants can either increase (HEC-2 with Al-B) or decrease (HEC-3 with Ti-B and HEC-4 with Fe-B) at *p* above 2 MPa, due to the possible influence of the heat release rate of reagents in the gas phase and changes in the HEC surface temperature.

## 4. Conclusions

The use of metal and metalloid UFPs in HECs makes it possible to regulate the *t_ign_* and the *u* for SPs, ensuring their complete combustion in the chamber. The nature of the metal and the protective layer on the metal particles affects their reaction efficiency in the oxidizing medium and the combustion intensity of the B particles, which allows them to realize their energy potential in the combustion chamber. The experimental study of the ignition and combustion of HECs containing AP, a butadiene rubber and ultrafine Me-B mixture fuel demonstrates the effect of changing the exponent *n* in the correlations with the *t*_ign_(*q*) and *u*(*p*) for SPs.

Replacing Alex with Al-B UFP (mass ratio of 55.5/44.5%) in HEC-2 reduced the *t_ign_* by 6–16% over the heat flux density range of 65–100 W cm^−2^ and increased the *u* by 7–18% over the pressure range of 0.5–5.0 MPa due to the increase in the heat release rate near the sample surface during the joint combustion of the Al and B particles. The exponent *n* in the *u*(*p*) correlation was at the maximum and was equal to 0.81 (80% more than that of HECs with AlB_2_ micron powder).

The use of Ti-B UFP (mass ratio of 68.9/31.1%) in HEC-3 reduced the *t*_ign_ by 20–27% (over the heat flux density range of 65–210 W cm^−2^) compared to the base HEC-1 with Alex. Micro-explosive and rapid combustion of Ti particles promoted the thermal oxidation of the B particles and the development of flame processes with increasing temperature and gas outflow rate from the HEC-3 surface. The *u* increased by 10–55% over the pressure range of 0.5–1.5 MPa and decreased by 6–24% over the pressure range of 2.5–5.0 MPa due to decreasing the exponent *n* to 0.45.

In the case of using Fe-B UFP (mass ratio of 83.7/16.3%) in HEC-4, the *t_ign_* had similar values (difference was less than 3%) in comparison to the *t*_ign_ of HEC-1 with Alex. Iron nano-sized particles catalyzed the thermal decomposition of AP and increased the outflow rate of gas decomposition products from the sample surface, forming a flame zone with the maximum length. Additional heat release during the oxidation of the B particles in the flame increased the rate of chemical reactions and combustion stability. The exponent *n* in the *u*(*p*) correlation was at the minimum and was equal to 0.25.

## Figures and Tables

**Table 1:** Calculated kinetic parameters of HEC ignition.

HECs	*E*, kJ mol^−1^	*Q* · *z*, W g^−1^	*T*_ign_ *, K at *q* = 60–200 W cm^−2^	*W* **, kW g^−1^ at *T*_ign_
1. Alex	119.0	1.82 · 10^16^	503–546	7.9–75.0
2. Al-B	56.8	5.17 · 10^9^	490–575	4.6–35.7
3. Ti-B	57.0	1.51 · 10^10^	461–536	5.3–42.1
4. Fe-B	81.2	1.58 · 10^12^	503–566	5.8–50.7

* *T*_ign_ = 0.287 · *q* · (*t*_ign_/(*λcρ*))^0.5^ + *T*_0_; ** *W* = *Q* · *z* exp (–*E*/*RT*).

## Data Availability

Data are contained within the article.
